# The effects of chemotherapy on primary small bowel cancer: A retrospective multicenter observational study in Japan

**DOI:** 10.3892/mco.2013.150

**Published:** 2013-07-22

**Authors:** TSUNEKAZU MIZUSHIMA, HIROSHI TAMAGAWA, HIDEYUKI MISHIMA, KIMIMASA IKEDA, SHIGEO FUJITA, HIROKI AKAMATSU, MASAKAZU IKENAGA, TADASHI ONISHI, MUTSUMI FUKUNAGA, TAKAYUKI FUKUZAKI, JUNICHI HASEGAWA, ICHIRO TAKEMASA, MASATAKA IKEDA, HIROFUMI YAMAMOTO, MITSUGU SEKIMOTO, RIICHIRO NEZU, YUICHIRO DOKI, MASAKI MORI

**Affiliations:** 1 Clinical Study Group of Osaka University (CSGO), Colorectal Group, Osaka, Japan; 2 Department of Surgery, Osaka University Graduate School of Medicine, Osaka, Japan; 3 Department of Surgery, Osaka General Medical Center, Osaka, Japan; 4 Unit of Cancer Center, Aichi Medical University, Aichi, Japan; 5 Department of Surgery, Minoh City Hospital, Osaka, Japan; 6 Department of Surgery, Social Insurance Kinan Hospital, Wakayama, Japan; 7 Department of Surgery, Osaka Police Hospital, Osaka, Japan; 8 Department of Surgery, National Hospital Organization, Osaka National Hospital, Osaka, Japan; 9 Department of Surgery, NTT West Osaka Hospital, Osaka, Japan; 10 Department of Surgery, Sakai City Hospital, Osaka, Japan; 11 Department of Surgery, Ikeda City Hospital, Osaka, Japan; 12 Department of Surgery, Osaka Rosai Hospital, Osaka, Japan

**Keywords:** small bowel cancer, palliative chemotherapy, adjuvant chemotherapy, retrospective chart review

## Abstract

Small bowel cancer is relatively rare among gastrointestinal tract cancers, including esophageal, gastric and colorectal cancers. The majority of cases of small bowel cancer are diagnosed at an advanced stage, resulting in poor outcomes. The clinical effects of chemotherapy on small bowel cancer have been investigated in a limited number of studies from Europe and the USA. However, they have not yet been fully investigated in Asian countries, including Japan. This retrospective multicenter observational study was designed to investigate the efficacy of chemotherapy on small bowel cancer. A questionnaire survey was conducted in 28 hospitals affiliated with the Osaka University Hospital. We retrospectively reviewed the medical records of 61 patients with small bowel cancer (32 patients who were unable to undergo curative resection or had unresectable distant metastases and 29 who underwent curative resection), treated between 1996 and 2009, to evaluate the outcomes and the efficacy of chemotherapy. There was no significant difference in the overall survival between the patients undergoing curative resection with postoperative adjuvant chemotherapy and those without postoperative adjuvant chemotherapy. In patients with non-curative resection or unresectable distant metastases, the response rate to chemotherapy was 31.6% and the overall survival was significantly higher compared to that without chemotherapy (P=0.008). The study results suggested that chemotherapy is effective for Japanese patients with small bowel cancer who cannot undergo curative resection or have unresectable distant metastases.

## Introduction

Small bowel cancer is relatively rare among gastrointestinal tract cancers, such as esophageal, gastric and colorectal cancers (1). The recent advent of capsule endoscopy and double-balloon enteroscopy (2) have expanded the range of examinations available to evaluate lesions in the small intestine. However, screening for small bowel cancer is still not routinely performed. Accordingly, the majority of cases are diagnosed at an advanced stage and, therefore, have a poor prognosis (1).

Several large phase III studies of gastric and colorectal cancers have demonstrated that chemotherapy may prolong survival in patients who are not able to undergo curative resection or have unresectable distant metastases. Furthermore, adjuvant chemotherapy may improve relapse-free and overall survival in patients undergoing curative resection. However, in contrast to other gastrointestinal tract cancers, such as gastric and colorectal cancer, the clinical significance of chemotherapy for small bowel cancer has not yet been fully investigated. This is likely due to difficulties in conducting prospective clinical studies that include a sufficient number of small bowel cancer patients. This retrospective multicenter observational study was designed to examine the efficacy of chemotherapy on small bowel cancer.

## Materials and methods

### Patient records

A questionnaire survey was conducted in 28 hospitals affiliated with Osaka University Hospital. The medical records of 61 patients with small bowel cancer, treated between 1996 and 2009, were retrospectively reviewed. Of these patients, 32 were unable to undergo curative resection or had unresectable distant metastases and 29 underwent curative resection. Palliative chemotherapy was prescribed for 19 of the 32 patients with non-curative resection or unresectable distant metastases, and postoperative adjuvant chemotherapy was administered to prevent recurrence in 14 of the 29 patients undergoing curative resection. Clinicopathological characteristics were assessed in all the patients, including comorbidities associated with the onset of small bowel cancer, such as Crohn’s disease and hereditary non-polyposis colorectal cancer (HNPCC) and the presence/absence of multiple primary cancers. We also evaluated the recurrence rate and overall survival in patients undergoing curative resection with/without postoperative adjuvant chemotherapy and the response rate to palliative chemotherapy and overall survival with/without chemotherapy in patients who were unable to undergo curative resection or had unresectable distant metastases.

This retrospective study was approved by the Institutional Review Board and informed consent was waived.

### Statistical analysis

The comparison between means was performed using the Student’s t-test and the proportions were compared using the Chi-square test. The survival rate was calculated using the Kaplan Meier method and the groups were compared using the log-rank test. P≤0.05 was considered to indicate a statistically significant difference.

## Results

### Characteristics of patients undergoing non-curative resection

The group of patients who underwent non-curative resection or had unresectable distant metastases included 17 men and 15 women (median age 64 years; range, 26–88 years). One patient had comorbid Crohn’s disease related to the onset of small bowel cancer and no patients had HNPCC. Metachronous multiple primary cancers occurred in the stomach in 2 patients, the rectum in 1 patient and the breast in 1 patient. The small bowel cancer was located in the jejunum in 21 patients and the ileum in 11 patients. All patients had undergone surgery and the postoperative TNM stage was III in 1 patient and IV in 31 patients. The surgical curability types were R1 in 5 patients and R2 in 27 patients. The first-line chemotherapy regimen was either 5-fluorouracil (5-FU)/S-1 [plus cisplatin (CDDP)] (n=9 patients), FOLFOX/CapeOX (n=6) or other regimens (n=4) (Table I).

### Characteristics of patients undergoing curative resection

The group of patients undergoing curative resection included 16 men and 13 women (median age 65 years; range, 26–81 years). There were no comorbidities related to the onset of small bowel cancer in this group. Metachronous multiple primary cancers occurred in the colon in 2 patients, the duodenum in 1 patient and the bladder in 1 patient. The small bowel cancer was located in the jejunum in 16 patients and the ileum in 13 patients. All the patients underwent complete resection (surgical curability, R0). The postoperative TNM stage was I in 3 patients, II in 14 patients and III in 12 patients. The adjuvant chemotherapy regimen was UFT/S-1, 5′DFUR/capecitabine or other regimens (Table II).

Due to the retrospective multicenter study design, detailed reasons for performing or not performing palliative/adjuvant chemotherapy, as well as for selecting a particular regimen, could not be ascertained. However, there were no significant differences in patient background factors between the two groups.

### Therapeutic effects

The therapeutic effect of palliative chemotherapy in 19 of the 32 patients with non-curative resection or unresectable distant metastases was as follows: 0 patients, complete response; 6, partial response; 5, stable disease; 5, progressive disease; and 3, not evaluable. The response rate was 31.6% (6/19) (Table III).

Of the 29 patients who underwent curative resection, recurrence was observed in 4 of the 15 patients without postoperative treatment and in 4 of the 14 who received postoperative adjuvant chemotherapy, demonstrating no statistically significant difference.

### Overall survival

**Figure 1:**
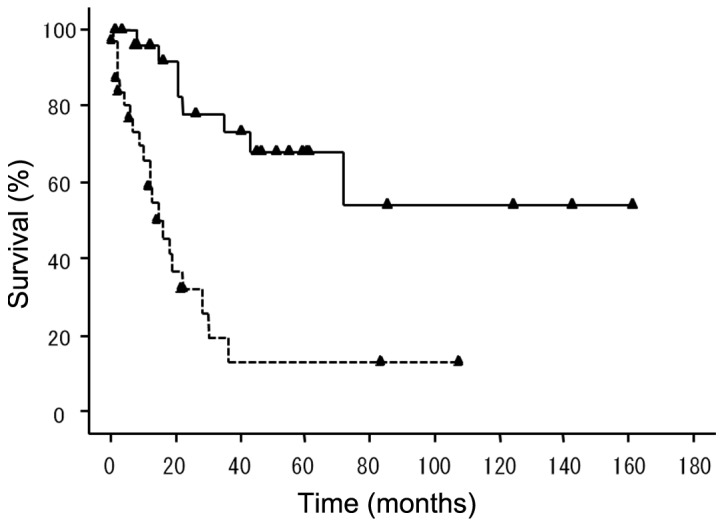
Overall survival among patients with non-curative resection or unresectable distant metastases (n=32, solid line) vs. patients undergoing curative resection (n=29, dotted line). The overall survival rate was 73.1% at 3 years and 67.9% at 5 years for patients undergoing curative resection, compared to 19.2 and 12.8%, respectively, in patients who were unable to undergo curative resection or had unresectable distant metastases, indicating significantly poorer outcomes in the latter group of patients (P<0.0001).

The overall survival rate was 73.1% at 3 years and 67.9% at 5 years in patients undergoing curative resection and 19.2 and 12.8%, respectively, in those who were unable to undergo curative resection or had unresectable distant metastases, indicating significantly poorer outcomes in the latter group of patients (P<0.0001) (Fig. 1).

**Figure 2:**
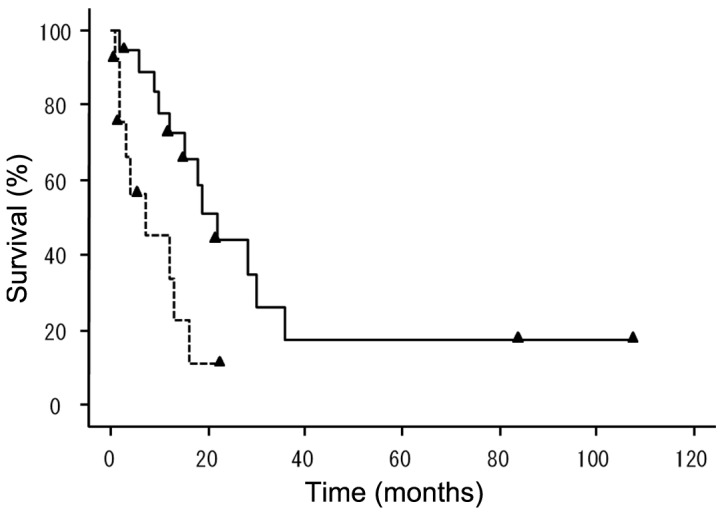
Overall survival among patients with non-curative resection or unresectable distant metastases with postoperative chemotherapy (n=19, solid line) vs. those without chemotherapy (n=13, dotted line). Among patients with non-curative resection or unresectable distant metastases, the 3-year overall survival rate was 26.3% with chemotherapy, compared with 13.8% without chemotherapy (P=0.008).

**Figure 3:**
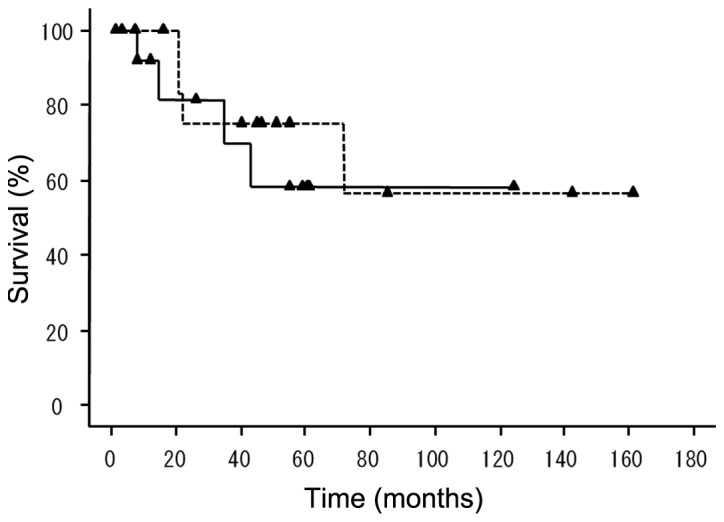
Among patients who underwent curative resection, there was no significant difference in the overall survival between patients who were administered postoperative adjuvant chemotherapy (n=14, solid line) and patients who were not (n=15, dotted line).

In patients with non-curative resection or unresectable distant metastases, the 3-year overall survival rate was significantly higher when compared to without chemotherapy (26.3 vs. 13.8%, respectively; P=0.008; Fig. 2). However, among patients who underwent curative resection, there was no significant difference in the overall survival between those who received and those who did not receive postoperative adjuvant chemotherapy (Fig. 3).

## Discussion

In this retrospective multicenter study, we investigated the effects of two different types of chemotherapy on patients with small bowel cancer: palliative chemotherapy for patients who were unable to undergo curative resection or had distant metastases and adjuvant chemotherapy for those who underwent curative resection.

The prevalence of small bowel malignancies is low, accounting for <5% of gastrointestinal tract cancers. Approximately 30–50% of these malignancies are adenocarcinomas, with a progressively increasing incidence (3). However, few studies have reported treatment outcomes in a large patient population. The few studies that have focused on this issue are mostly from Europe and the United States of America (USA), whereas the number of such studies from Asian countries, including Japan, is limited. Studies conducted in Europe and the USA have reported that the median survival of patients with small bowel cancer is ~20 months and the overall survival at 5 years is ~30%; these figures have remained unchanged for the last 20 years (1,4).

Several novel strategies for the treatment of gastric (5) and colorectal (6,7) cancers have been developed and evaluated based on the results obtained from large clinical studies and have led to significantly improved outcomes for patients receiving chemotherapy. However, as the number of patients with small bowel cancer is limited, chemotherapeutic regimens that are effective in gastric and colorectal cancers are used to treat small bowel cancer in the clinical setting. Unlike gastric and colorectal cancer, the outcomes of chemotherapy for small bowel cancer have been reported in only a limited number of studies from Europe and the USA. Accordingly, no standard treatment has been established.

Previous studies have reported the benefits of a combination of 5-FU agents and platinum-based chemotherapeutic agents, such as oxaliplatin, for first-line treatment, with a response rate of 30–50% and an overall survival of 14.8–20.4 months, which are outcomes similar to those of advanced or recurrent colorectal cancer (8–10). Although the efficacy of folinic acid plus 5-FU plus irinotecan (FOLFIRI), a regimen originally designed for colorectal cancer, as second-line chemotherapy for small bowel cancer following failure of first-line platinum-based chemotherapy has been reported, the outcomes of FOLFIRI were unsatisfactory: the response rate was 20%, the progression-free survival was 3.2 months and the overall survival was 10.5 months (11). A high percentage of small intestinal tumors reportedly express epidermal growth factor receptor and vascular endothelial growth factor, suggesting that molecular-targeted therapy may improve the outcomes (12).

The clinical studies evaluating outcomes in Europe and the USA commonly include patients with duodenal cancer. Although several studies (10) have reported that chemotherapy is more effective in treating duodenal compared to jejunal or ileal cancer, no consensus has been reached as yet. In addition, patients with duodenal cancer often require highly invasive surgery, including pancreaticoduodenectomy. Therefore, the conditions of those patients receiving chemotherapy may be different from those of patients with jejunal or ileal cancer. Taking these factors into account, patients with duodenal cancer were excluded from this study and the effects of chemotherapy were only investigated in those with jejunal or ileal cancer.

In the present study, the first-line chemotherapy regimens used for patients who were unable to undergo curative resection or had unresectable distant metastases were 5-FU/S-1 (plus CDDP), which was based on chemotherapy for gastric cancer, and the FOLFOX/CapeOX regimen, which was based on chemotherapy for colorectal cancer. The response rate of 31.6% and the median survival of 22 months were similar to those reported by previous studies (8–10) involving patients with advanced or recurrent cancer in Europe and USA.

The number of studies on adjuvant chemotherapy for small bowel cancer is limited and the effects of chemotherapy on the prevention of recurrence and prolongation of survival remain to be verified in small bowel cancer patients (13,14). Our study results did not confirm the efficacy of postoperative adjuvant chemotherapy. Although adjuvant chemotherapy for colorectal cancer may reduce the recurrence rate and marginally improve overall survival, the detection of such improvements generally requires meta-analyses or comparisons that involve a large number of patients (15,16). Results of the present study do not rule out the effectiveness of postoperative adjuvant chemotherapy in patients with small bowel cancer undergoing curative resection. However, they emphasize the need for future studies comprising larger patient samples.

In conclusion, due to the retrospective design and the limited number of patients, our study results must be interpreted with caution. Our findings indicate that chemotherapy may be effective for Japanese patients with small bowel cancer who are not considered appropriate candidates for curative resection or who have distant metastases. Future studies are required to investigate optimal therapeutic regimens and evaluate the efficacy of postoperative adjuvant chemotherapy in a larger number of patients.

## Figures and Tables

**Table I:** Characteristics of patients with non-curative resection or unresectable distant metastases (n=32).

Variables	n	With chemotherapy (n=19)	Without chemotherapy (n=13)
Median age (range)	64 (26–88)	62 (30–86)	66 (26–88)
Gender			
Male	17	11	6
Female	15	8	7
Comorbidity			
Crohn’s disease	1	1	-
HNPCC	-	-	-
Multiple primary cancers			
Gastric	2	2	-
Rectal	1	1	-
Breast	1	1	-
Location			
Jejunum	21	13	8
Ileum	11	6	5
TNM stage			
III	1	1	0
IV	31	18	13
Surgical curability			
R1	5	3	2
R2	27	16	11
Regimen			
5-FU/S-1 (+CDDP)		9	-
FOLFOX/CapeOX		6	-
Other regimens		4	-

HNPCC, hereditary non-polyposis colorectal cancer; 5-FU, 5-fluorouracil.

**Table II:** Characteristics of patients undergoing curative resection (n=29).

Variables	n	With chemotherapy (n=14)	Without chemotherapy (n=15)
Median age (range)	65 (26–81)	55 (26–75)	70 (48–81)
Gender			
Male	16	6	10
Female	13	8	5
Comorbidity			
Crohn’s Disease	-	-	-
HNPCC	-	-	-
Multiple primary cancers			
Colon cancer	2	1	1
Duodenal cancer	1	-	1
Bladder cancer	1	-	1
Location			
Jejunum	16	9	7
Ileum	13	5	8
TNM stage			
I	3	0	3
II	14	5	9
III	12	9	3
Regimen			
UFT/S-1		6	-
5′DFUR/capecitabine		4	-
Other regimens		4	-

HNPCC, hereditary non-polyposis colorectal cancer.

**Table III:** Response rates.

Response	Therapeutic effect	5-FU/S-1 (+CDDP) (n=9)	FOLFOX/CapeOX (n=6)	Others (n=4)
CR	-	-	-	-
PR	6	3	2	1
SD	5	2	2	1
PD	5	2	1	2
NE	3	2	1	-

CR, complete response; PR, partial response; SD, stable disease; PD, progressive disease; NE, not evaluated.
